# Restorative dentistry in Early Modern Scotland: archaeological evidence of the use of a gold ligature

**DOI:** 10.1038/s41415-025-9107-3

**Published:** 2026-04-24

**Authors:** Jenna M. Dittmar, Rebecca Crozier, Ali Cameron, Bruce Mann, Marc F. Oxenham

**Affiliations:** 41415549660001 Biomedical Affairs, Edward Via College of Osteopathic Medicine, Louisiana, Monroe, LA, USA; Department of Archaeology, University of Aberdeen, Aberdeen, UK; 41415549660002 Department of Archaeology, University of Aberdeen, Aberdeen, UK; 41415549660003 Cameron Archaeology, Aberdeen, UK; 41415549660004 Aberdeenshire Council Archaeology Service, Aberdeen, UK; 41415549660005 Department of Archaeology, University of Aberdeen, Aberdeen, UK; School of Archaeology and Anthropology, Australian National University, Canberra, Australia

## Abstract

The earliest known example of restorative dentistry from Scotland is described. A middle-aged adult male, who lived between 1460–1670 CE in Aberdeen, was observed to have a gold ligature fixed to the right lateral and left central mandibular incisors, forming a bridge for the potentially missing right central incisor. As this individual lived before the establishment of dentistry as a profession during the 19^th^ century, the ligature was likely placed by a semi-skilled practitioner, such as a jeweller, barber, or dentatore. The archaeological and documentary evidence indicates he was a relatively wealthy member of the community based on his prestigious burial location, and that he was able to afford this type of dental work. Given the social importance of an individual's appearance during the Late Medieval and Early Modern era as an outward expression of their moral character, it is likely the rationale for undergoing this procedure extended beyond retaining masticatory abilities and oral function.

## Key points

- Understanding pre-modern dentistry: demonstrates that dental practices existed long before the establishment of modern dentistry, which sheds light on the evolution of dental care.
- Cultural context: highlights how physical appearance and dental health were tied to perceived moral character in historical societies, offering a deeper understanding of past social dynamics.
- Social inequalities in health: illustrates that wealthier individuals had access to advanced dental treatments, demonstrating how access to healthcare and cosmetic procedures was historically linked to socio-economic status.
- Evidence of pre-modern medical practices: adds to the growing body of evidence about early medical practices, which will contribute to future historical interpretations of health care in the past.

## Introduction

While many archaeological studies show that poor oral health was the norm in the pre-modern era,^1^^,^^2^^,^^3^ the importance of maintaining and caring for one's teeth has long been recognised with numerous risk factors associated with poor oral health.^4^^,^^5^^,^^6^^,^^7^ This is evidenced by an accumulating, though relatively scant, body of documentary and archaeological evidence that predates the establishment of dentistry as a profession during the 19^th^ century.^8^^,^^9^^,^^10^^,^^11^^,^^12^^,^^13^^,^^14^^,^^15^^,^^16^ Here we add to this corpus of evidence by presenting the earliest known case of the use of a dental ligature in Scotland.

## Dental ligature: a brief history

The origins of restorative dentistry, defined as the repairing or replacing of damaged or missing teeth, is controversial. Archaeological evidence suggests that restorative dentistry was practised during the Neolithic (early farming) period, although some argue that such practices may have predated the Neolithic period. Oxilia *et al.*^17^ report a possible example of therapeutic modification of a carious lesion in the lower right third molar on an individual approximately 14,000 years ago during the Late Upper Palaeolithic period. Another example of therapeutic drilling comes from Neolithic Pakistan.^13^ The earliest known dental fillings also date to this time period. Bernardini *et al.*^12^ report the presence of beeswax inside a carious lesion located on a canine tooth from a 6,500-year-old individual from Slovenia. Other possible examples of dental fillings have been reported from the New Kingdom (1550–1070 BCE)^8^ and the Ptolemaic periods (305–30 BCE) in Egypt. ^15^^,^^16^

The earliest accepted examples of dental ligatures occur in Egypt.^10^^,^^11^^,^^18^ Silver and gold wires are present on each of the four known dental bridges, employed to stabilise loose teeth, or replaced a tooth that was lost. The first published example was reported by Junker^19^ following the excavation of the Western cemetery of Cheops' pyramid. He observed two mandibular molars connected by gold wires.^18^ Though initially thought to have been used during life, subsequent assessments suggest that the gold wires were more likely placed post-mortem as part of the mummification process.^10^ Similarly, the reassessment of the dental work recovered from el-Quatta, near Cairo (c. 2,500 BCE), suggest that these may have also been placed post-mortem.^10^ The earliest clear dental prosthetic is a bridge that was found during the 1952 excavation at Tura el-Asmant and dates to the Ptolemaic period (305–30 BCE).^10^^,^^11^ This bridge was fixed into place with a silver wire that passed through two drilled holes on the mesiodistal aspect of the crown of the right maxillary central incisor.

Other examples where a ligature was used to secure and stabilise replacement teeth made from a wide variety of materials have been observed in Phoenician (c. 1200–330 BCE), Etruscan (c. 900–200 BCE), and Roman (27 BCE–476 CE) contexts.^20^^,^^21^^,^^22^^,^^23^^,^^24^^,^^25^ However, the current consensus is that many of the Etruscan examples were decorative replacements worn following tooth ablation.^21^^,^^22^ Other examples were likely crafted to be fitted after death to ensure that the body was ‘complete' before burial, rather than as a form of dental care.^26^

By the Middle Ages in Europe (500–1500 CE), ligating a loose tooth using silver or gold wire was described in a range of medical treatises by several prominent physicians. One of the first such descriptions was included by Abu Al Qasim Al Zahrawi (latinised as Abulcasis/Albucasis) in his ‘*De Chirurgia*' in c. 1000 CE. This method was perpetuated by several subsequent authors of medical texts. In Guy de Chauliac's influential work, *Inventarium seu collectorium in parte cyrurgicali medicine* (also known as the *Chirurgia magna*), written in 1363 CE, he advised that if teeth became loose, that they be tied to healthy teeth using a gold chain ‘after the manner of Abulcasis.' He also recommended the use of a fine ligature to secure in place replacement teeth taken from another person or artificial teeth made of ox bone.^27^

During the Middle Ages, there was an increase in the production of medical, surgical and scientific treatises which often contained remedies for various oral afflictions.^28^ The second half of the 14^th^ century saw an explosion in vernacular translations of such treatises. Though surgeons and barbers were the target audience for most surgical publications, some translations of medical treatises appear to have been intended for ‘an audience beyond that of scholars and medical practitioners'.^28^ For example, Lanfrank's treatise entitled ‘Science of chirurgie' that was originally written in Latin in c. 1295 was translated into Middle English c. 1380 CE and Guy's *Chirurgia magna* was translated into French and other vernacular languages in 1478 CE.^28^

Most of these surgical texts are relatively brief in their instruction regarding teeth and oral health more generally. One of the reasons that has been suggested for this is that teeth were generally considered outside the purview of medieval physicians and surgeons. During the Middle Ages, teeth were often treated by barbers, or dentatores, who were individuals that specialised in teeth.^29^ The first book dedicated entirely to the practice of dentistry was published in 1530 in Germany, titled *Artzney buchlein* or ‘*The little medicinal book for all kinds of diseases and infirmities of the teeth*'.^30^

Though these texts were widely circulated in the medieval and Early Modern world, few examples of dental ligatures have been found at archaeological sites in Europe that predate the 17^th^ century. The use of gold ligatures has been previously reported from 15th century Portugal,^31^ and in 16^th^ to early 17^th^ century France.^32^ Here we add to this corpus of evidence by presenting the earliest known case of the use of a dental ligature in Scotland. Duffy *et al.*^33^ first made reference to a gold wire or dental bridge attached to the mandibular anterior teeth of an adult male who was buried inside a parish church in Aberdeen. The aim of this paper is to contextualise this finding within the larger body of documentary and archaeological evidence to further enhance our understanding of the measures taken by past people to maintain their oral health.

## Materials and methods

**Fig. 1:**
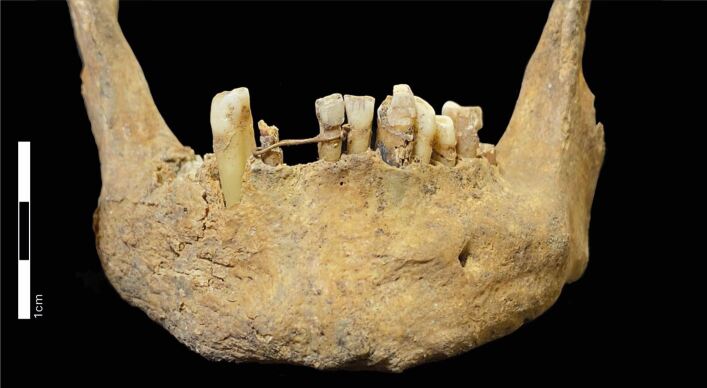
Gold ligature surrounding the left central incisor and the right lateral incisor on the mandible of an adult male buried in the East Kirk of the parish church of St Nicholas, Aberdeen, Scotland. Photograph by Jenna Dittmar

The mandible that is the focus of this paper was recovered during a salvage excavation before the redevelopment of the East Kirk of St Nicholas Kirk in Aberdeen, Scotland (Fig. 1). The earliest documentary evidence for the Kirk of St Nicholas occurs in 1157 CE,^34^ but an earlier origin in the 11^th^ century it is suspected.^35^ As such, the church served as the main location for Christian worship in the medieval burgh for around 900 years. Rebuilding and expansion of the kirk occurred through the 14−16^th^ centuries when it was recognised as one of the largest churches in Scotland.^34^ When the Protestant Reformation (1534−1603 CE) reached Scotland in 1560 CE, the Catholic phase of the kirk ended. This change was denoted with the appointment of the first Protestant Minister, Adam Heriot, to the Kirk of St Nicholas in 1560 CE.^36^ From January to December 2006, an archaeological excavation was undertaken inside the East Kirk by the Aberdeen City Council Archaeological Unit. The skeletal remains of around 900 individuals, along with 3.5 metric tonnes of disarticulated skeletal material were excavated.^35^

As part of a larger research project focused on temporal health trends throughout Scotland, the skeletons salvaged from the Kirk of St Nicholas were reassessed, including the mandible and assumed gold ligature. The biological sex of the individual was determined using sexually dimorphic features of the mandible,^37^^,^^38^^,^^39^ while age-at-death was estimated based on dental development^40^ and the degree of occlusal tooth wear.

As this element was disarticulated and thus divorced from its original context, a fragment of the mandible was radiocarbon dated at the Scottish Universities Environmental Research Centre radiocarbon laboratory, following their standard procedures.^41^ Analysis was undertaken using OxCal versus 4.4^42^^,^^43^ and calibrated using a mix of the IntCal20 and Marine20 calibration curves.^44^^,^^45^ A regional marine offset (ΔR) of -150 ± 52 years was used in the calibration.

### Description of element

The mandible of interest was approximately 70% complete: the posterior portion of the left ramus was not preserved. In the mandibular dental arcade, nine teeth remained *in situ,* four were lost post-mortem, and one (the right central incisor) was lost ante-mortem. The third molars were not present, nor was there any observable evidence to suggest that they had been lost ante- or post-mortem. It is not known if the third molars ever formed or erupted. The overall size of the element and the stage of dental development for the present teeth (complete), indicates that the mandible in question is from an adult (defined as over 18 years of age) at the time of their death. The degree of occlusal wear is consistent with other middle-aged individuals from the Kirk that have been aged independently of their dentition. Based on the observable sexually dimorphic features, it is likely that the mandible was from a male individual.

An examination of the teeth reveals that this individual had poor oral health. Carious lesions were present on multiple teeth; gross carious lesions where more than 50% of the crown was destroyed were evident on three teeth (the right lateral incisor, as well as the left first and second molar). Minor calculus deposits were present on all remaining teeth. No periapical cysts or abscesses were observed. Unfortunately, the full extent of the oral health issues that this individual experienced cannot be determined due to post-mortem damage and the subsequent loss of teeth.

**Fig. 2:**
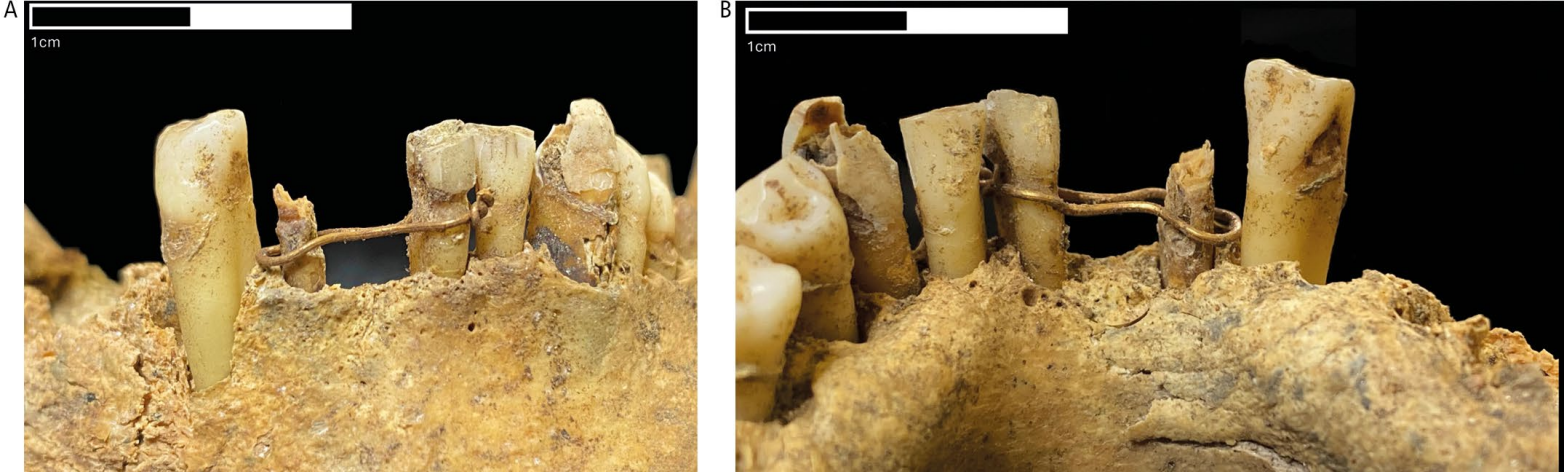
Close up of gold ligature from the (A) labial and (B) lingual aspects. Photographs by Jenna Dittmar

The most noteworthy feature on this element is the presence of fine gold wire (see below for details) that encircled the right lateral incisor and the left central incisor, spanning the gap between these two teeth where the right central incisor would be (Fig. 1, Fig. 2). This wire is located around the dental cervix of the aforementioned teeth and is held in place with a tightening knot located on the labiodistal aspect of the left central incisor. There is a marked depression that extends along the dental cervix on the labial and lingual aspects of the left central incisor that was likely caused by prolonged rubbing of the wire against the tooth root. This suggests that the ligature was likely in place for a substantial period of time before this individual died. The shape of the wire as it extends across the healed socket for the right central incisor suggests that this ligature was placed after the loss of this tooth. This being the case, the most likely purpose for this ligature was to attempt to either retain the right lateral incisor or to provide a bridging scaffold to sustain a prosthetic tooth.

### Composition of the ligature

**Fig. 3:**
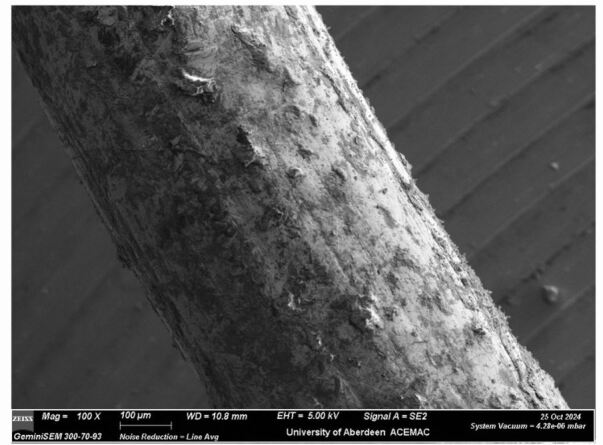
Note tool marks (longitudinal grooves) caused by holes in the draw plate used to manufacture the wire

**Fig. 4:**
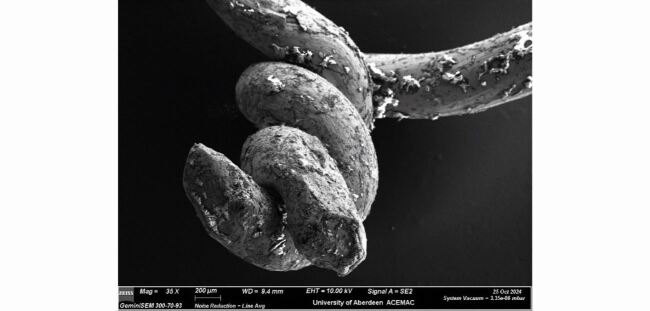
35x magnification of the knotted end of the ligature

Scanning electron microscopy with an energy dispersive x-ray spectroscopic assay of the wire was carried out with a semi-quantitative analysis, indicating the ligature was an alloy made up of approximately (mean of six separate scan sites) 82.4% gold, 9.8% silver, and 2.5% copper, with the remaining measurable elements including sulphur, aluminium and oxygen. This would be considered 20 carat gold. Figure 3 shows longitudinal furrows in a section of the ligature at 100x magnification. These furrows are associated with the manufacture of the wire, which would have been pulled through a series of graduated holes in a draw plate (for further discussion see).^46^ Figure 4 illustrates a 35x magnification of the knotted end of the ligature which shows flattening on the end, presumably due to the use of a pair of pilers.

### Date of element

The radiocarbon date indicates that this individual most likely died between 1460–1670 cal CE, 95.4% range (381 ± 23 radiocarbon age before present, GU59616).

## Discussion

The date of this individual indicates that this procedure was performed before the establishment of dentistry as a profession during the 19^th^ century. Though the first recognised dental qualification in the UK was introduced in 1860, dentistry was practiced by many skilled (and unskilled) practitioners before this.^47^^,^^48^ Those in need sought treatment from barbers, barber-surgeons, dentatores (who were individuals that specialised in teeth), or from other healers or craftsmen in their communities. Depending on availability, one could also seek relief from a ‘tooth-drawer', who were often carnival performers that travelled around the country peddling proprietary methods for ‘painlessly' extracting teeth.^49^

Within early modern Scottish communities, the administration of most health care was undertaken by local women who would pull teeth, as well as provide herbal medicine, prayers and charms.^50^ Various remedies for oral-health related concerns have been identified in written sources.^51^ Local treatment for toothache on the Isle of Skye included green turf heated with embers applied to site of pain.^51^ Accounts from Aberdeen indicate that one local treatment for abscesses (as well as eczema) was a cow dung poultice.^51^ The administration of such folk remedies was practiced in Scotland into the 20^th^ century.^52^

For certain ailments, specialist materials or appliances were required. As is seen in this case, gold alloy wire was, and still is, a preferred material in restorative and conservative dentistry due to its resistance to corrosion and tarnishing and biocompatibility.^53^ Given the materials involved a 20-carat gold, silver and copper alloy, a goldsmith was most likely sought out to craft and possibly even fit the ligature. Between 1460–1670 CE, at least 22 goldsmiths were operating in Aberdeen.^54^ Any number of these craftsmen were likely capable of producing a simple gold wire (as described above) and creating the observed tightening knot.

Of the 100 individuals that were excavated from inside the East Kirk of St Nicholas that dated to the Early Modern period, the individual presented here was the only one with clear evidence of dental work. The rarity of this find indicates that such procedures were out of reach for most of the inhabitants of Early Modern Aberdeen. It is likely that the cost of the gold was a barrier to most. It is also possible this individual had this work done elsewhere. Unfortunately, there is no way to be certain of this.

The underlying reasons for undergoing this procedure were likely multifaceted. During the Late Medieval and Early Modern period, an individual's physical appearance was believed to be indicative of their individual character.^55^ The appearance of a person and their perceived health was linked to one's sins.^56^ As such, the social importance of an individual's smile encouraged those who were able to afford such treatments to seek them out.^49^

## Conclusions

This paper presented the earliest known example of restorative dentistry in Scotland: the use of a gold alloy ligature either to stabilise an unstable mandibular incisor or provide a bridging structure to facilitate the fitting of a prosthetic tooth. This case study contributes to a growing body of evidence for the pre-modern practice of restorative dentistry. As this individual lived before the establishment of dentistry as a profession during the 19^th^ century, the ligature was likely placed by a semi-skilled practitioner, who may also have been the source of the gold wire: a jeweller. Though it was not possible to determine where this individual would have received this treatment, the location of burial (i.e., inside the East Kirk of an affluent parish church) indicates he was a relatively wealthy member of the community. Given the social importance of one's appearance during the Late Medieval and Early Modern era, as an outward expression of their moral character, it is likely the reasons for undergoing this procedure extended beyond retaining masticatory abilities and oral function.
